# Yellow fever vaccination coverage following massive emergency immunization campaigns in rural Uganda, May 2011: a community cluster survey

**DOI:** 10.1186/1471-2458-13-202

**Published:** 2013-03-07

**Authors:** James Bagonza, Elizeus Rutebemberwa, Malimbo Mugaga, Nathan Tumuhamye, Issa Makumbi

**Affiliations:** 1 School of Public Health, Makerere University College of Health Sciences, Kampala, Uganda; 2 Ministry of Health, Kampala, Uganda

**Keywords:** Yellow fever, Vaccination, Cluster survey, Evaluation, Pader

## Abstract

**Background:**

Following an outbreak of yellow fever in northern Uganda in December 2010, Ministry of Health conducted a massive emergency vaccination campaign in January 2011. The reported vaccination coverage in Pader District was 75.9%. Administrative coverage though timely, is affected by incorrect population estimates and over or under reporting of vaccination doses administered. This paper presents the validated yellow fever vaccination coverage following massive emergency immunization campaigns in Pader district.

**Methods:**

A cross sectional cluster survey was carried out in May 2011 among communities in Pader district and 680 respondents were indentified using the modified World Health Organization (WHO) 40 × 17 cluster survey sampling methodology. Respondents were aged nine months and above. Interviewer administered questionnaires were used to collect data on demographic characteristics, vaccination status and reasons for none vaccination. Vaccination status was assessed using self reports and vaccination card evidence. Our main outcomes were measures of yellow fever vaccination coverage in each age-specific stratum, overall, and disaggregated by age and sex, adjusting for the clustered design and the size of the population in each stratum.

**Results:**

Of the 680 survey respondents, 654 (96.1%, 95% CI 94.9 – 97.8) reported being vaccinated during the last campaign but only 353 (51.6%, 95% CI 47.2 – 56.1) had valid yellow fever vaccination cards. Of the 280 children below 5 years, 269 (96.1%, 95% CI 93.7 – 98.7) were vaccinated and nearly all males 299 (96.9%, 95% CI 94.3 – 99.5) were vaccinated. The main reasons for none vaccination were; having travelled out of Pader district during the campaign period (40.0%), lack of transport to immunization posts (28.0%) and, sickness at the time of vaccination (16.0%).

**Conclusions:**

Our results show that actual yellow fever vaccination coverage was high and satisfactory in Pader district since it was above the desired minimum threshold coverage of 80% according to World Health Organization. Massive emergency vaccination done following an outbreak of Yellow fever achieved high population coverage in Pader district. Active surveillance is necessary for early detection of yellow fever cases.

## Background

Yellow fever is an acute vaccine preventable viral haemorrhagic disease found mainly in tropical regions of Africa and America. It is caused by a Flavivirus which is transmitted by mosquito bites and has a natural reservoir in non-human primates. The virus is endemic in tropical areas of Africa and Latin America, with a combined population of over 900 million people [1]. The “yellow” in the name refers to the jaundice that affects some patients. Approximately 200,000 cases and 30,000 deaths occur annually especially in Africa [2] where the case-fatality rate ranges from 15–50% [3]. The yellow fever virus can cause devastating epidemics of potentially fatal, hemorrhagic disease. The disease can be confirmed through, serological testing by way of ELISA for yellow fever virus-specific IgM or isolation of the virus from blood samples. These are the recommended standard diagnostic tests for yellow fever although PCR tests which detect the viral genetic material have been used [1,4]. The number of yellow fever cases has increased over the past two decades due to declining population immunity to infection, deforestation, urbanization, population movements and climate change. This disease has no cure and treatment is symptomatic, aiming at reducing symptoms for the comfort of the patient and mass vaccination is the most important preventive measure [5]. Immunization of people at risk interrupts human to human transmission of the yellow fever virus especially when adequate vaccination coverage levels are reached. The vaccine is safe, affordable and highly effective, and appears to provide protection for 30–35 years or more [1]. The vaccine provides effective immunity within one week for 95% of persons vaccinated [6,7]. Despite mass vaccination campaigns to prevent and control these outbreaks, the risk of major yellow fever epidemics, especially in densely populated, poor urban settings, both in Africa and South America, has greatly increased [8]. Consequently, yellow fever is considered an emerging, or reemerging disease of considerable importance.

In December 2010, the Ugandan Ministry of Health (MOH) declared a Yellow fever outbreak based on nine laboratory confirmed samples from the five districts of Abim, Agago, Kitgum, Pader and Lamwo in northern Uganda. This part of Uganda, home to game reserves and national parks, borders South Sudan and Kenya where previous cases of yellow fever have been reported [9,10]. This was the largest ever recorded yellow fever outbreak in the country with an overall attack rate of 13 cases per 100,000 population based on confirmed cases [4]. A cumulative total of 181 confirmed cases with 45 deaths (CFR = 24.8%) were recorded, with no new cases reported since February 2011 [4,11]. The CFR among males (29.6%; 32/108) was nearly twice that of females (17.8%; 13/73) and the ages of suspected yellow fever cases varied from 3 months to 83 years with a mean age of 28.2 years and a standard deviation of 17.5 years [4]. Previous yellow fever outbreaks in Uganda had fewer or single cases [12,13]. For about 40 years now, no yellow fever cases had been reported in Uganda [14,15]. However, larger outbreaks had been reported in East Africa, with attack rates (cases per 100,000 persons) of 6,800 in Sudan [9] and 27.4 in Kenya [16]. In addition, the yellow fever vaccine is not part of the Expanded Program for Immunization (EPI) vaccines given routinely especially to children under five. It is mainly people travelling abroad from Uganda that are required to receive the yellow fever vaccine.

The Ugandan Ministry of Health developed a national response plan prioritizing surveillance and laboratory confirmation, case management, social mobilization and health education, and reactive vaccination to contain the outbreak. The national response team included Ministry of Health of Uganda, World Health Organization (WHO), Uganda Red Cross society (URCS), United Nations International Children Emergency Fund (UNICEF), and the U.S. Centers for Disease Control and Prevention (CDC). Others were African Field Epidemiology Network (AFENET), RESPOND (Responding to emerging pandemics and threats), MSF-Holland, Uganda National Expanded Program on Immunization (UNEPI) and World Vision. This campaign was conducted between the 22^nd^ and 26^th^ of January 2011 and targeted all persons aged 6 months and above in the affected districts. Approximately one million people were targeted in the affected region.

Pader district is found in northern Uganda and this region borders South Sudan which is prone to sporadic yellow fever outbreaks [17]. In this district, residents lead a primarily agricultural lifestyle, growing crops and raising cattle, sheep, and poultry. Community settlements near bushes and forests are common since most people have just returned from internally displaced peoples camps after the war in northern Uganda. They venture into the forests mainly for hunting and harvesting bamboo for sale. Potential mosquito-breeding sites include baobab trees with tree holes, rock holes, and a water reservoir on the outskirts of villages. The district has a projected population of 205,898 [18] and the yellow fever attack rate was 2.9 cases per 100 000 population with females being more affected than males.

During the response, 177 vaccination posts were created, 354 health workers and 590 volunteers from 295 villages were identified and trained. Vaccination posts were mainly health facilities (both public and private not for profit), outreach sites for routine EPI vaccines and a few schools. During the campaigns, Uganda Red Cross society (URCS) supported community-based activities such as social mobilization, transportation of vaccines and provision of logistics. The URCS volunteers also supported organization of beneficiaries, issuing of vaccination cards and other support activities at the various vaccination posts in this district. The target population for vaccination in this campaign was all persons aged 6 months and above with a target vaccination coverage of at least 80%. After the campaign, the reported or administrative yellow fever vaccination coverage was 75.9% and this was below the targeted 80% [11]. Administrative yellow fever vaccination coverages in the other districts were: Abim 120.5%, Agago 81.6%, Kitgum 77.6% and Lamwo 73.9% [11]. When the target coverage is not reached, the potential for an epidemic increases since there is a low prevalence of neutralizing antibodies to the yellow fever virus from previous vaccination or naturally acquired infection.

Underreporting of vaccine doses administered usually results in underestimation of the coverage. It is important to conduct coverage surveys to validate reported administrative coverages so as to inform imminent (mop-up activities) and future immunizations strategies. Probability-based surveys such as cluster surveys can estimate vaccination coverage even if the size of the target population is not known [19,20]. The aim of this survey was to estimate yellow fever vaccination coverage in Pader district and determine the reasons for non vaccination to recommend possible mop-up actions, guide future vaccination efforts and contribute to the control of yellow fever in the country.

## Methods

### Study design

We estimated the yellow fever vaccination coverage through a cluster survey based on the methodology recommended by World Health Organization for determining vaccination coverages of routine EPI vaccines [21]. Our study population was individuals aged nine months and above living in the Pader district at the time of the survey. We excluded the following from our study population: Individuals and children less than 5 years whose parents or caregivers were absent by the time of the survey. This survey was conducted 3 months after the massive emergency vaccination campaigns. We considered two main outcomes: vaccination status documented by the vaccination card (card only) and also by considering verbal reports of vaccination (card and history or recall). We only considered vaccination status assessed by cards and verbal reports from this campaign.

### Sample size calculation

The required sample size was 680 individuals with the following assumptions; a two-sided test with a precision of 0.05, 17 respondents per cluster, design effect of 2 [22], proportion of those with complete vaccinations based on the previous administrative coverage of 75.9% [11] and a non-response rate of 17% [23]. We needed 7 respondents from those below 5 years, 5 from those between 5 and 15 years and 5 from those above 15 years in each cluster. This survey was also used to assess vaccination coverage for routine EPI vaccines for children under five, the reason we needed 7 respondents from this age group based on the WHO 30 by 7 cluster sampling strategy [21].

### Sampling procedure

This post campaign evaluation of the yellow fever vaccination coverage was based on the WHO EPI coverage survey methodology using the cluster sampling approach [21]. In this method, villages were taken as clusters and the process involved two stages of sampling. A list of all villages containing registered voters was obtained from the district returning officer. The total population of a village was estimated based on the fact that the proportion of registered voters was approximately 43% in each village (this was the most recent population update data available following national elections that occurred in February 2011). The total population of Pader district was estimated by summing up all the number of people in each village. We then divided the total population of the district by 40 (the desired number of clusters) to obtain the sampling interval. A random start was obtained and proportionate to size probability sampling (PPS) was done to get all the 40 clusters systematically. The center of each cluster was located with the help of a local guide and we randomly determined the direction of movement throughout the cluster. We identified the first household randomly and then the next household to visit was the one nearest to the first one. In each cluster, a random sample of 17 individuals were selected taking seven from those below 5 years, five from those between 5–15 years and five from respondents above 15 years. Where we found more than one eligible respondents for the different age specific categories in a household, one respondent was selected by simple random sampling.

### Survey instruments

We used the modified WHO cluster survey forms for yellow fever vaccination for children and adults. The cluster survey tools were pretested in five villages which were not part of the study and were modified where necessary. The instrument sought the identity of respondents, awareness about the yellow fever vaccination campaign and yellow fever vaccination status and reasons for non vaccination. Most questions on the survey instruments were close ended and information on vaccination status was obtained from vaccination cards (where available) or from the respondent’s verbal history. For a vaccination card to be considered valid, it had to be dated and signed by appropriate Health authorities.

### Survey teams

The survey forms were administered by 30 trained interviewers who were supervised by two central and five district supervisors. Each team consisted of two to three interviewers who were proficient in both English and the local language (Acholi). Each district supervisor was responsible for two to three teams while the central supervisors monitored all the teams. The survey teams used vehicles to travel to the field and each team filled on average 10 survey forms daily (approximately one cluster per day). Face to face interviews were conducted with randomly selected participants. Where we found more than one eligible respondent in the household, one was selected by simple random sampling. For children below 5 years, a present adult household member was surveyed as a proxy regarding the child’s information. Data collection lasted five days and an individual was considered vaccinated if there was card evidence or through positive self reports. The supervisors and data collectors edited survey tools at the end of each day for completeness and consistency. Data were cleaned and stored on a daily basis by central supervisors. In order to check the accuracy of data entered, cross checking the print of a data set with a random number of survey forms picked from the sample was done.

### Data analysis

Data were coded and captured using Epi-Info 3.3.2 of 2005 version software and exported to STATA version 10.0 for analysis. Our main outcomes were measures of yellow fever vaccination coverage in each age-specific stratum, overall, and disaggregated by age and sex, adjusting for the clustered design and the size of the population in each stratum. The other outcomes were reasons for not receiving yellow fever vaccination.

### Ethical considerations

This survey was a programmatic research conducted in collaboration with the Ugandan Ministry of Health and WHO in response to an emergency public health outbreak (yellow fever) in northern Uganda. The Ministry of Health and the Uganda National Council for Science and Technology consider outbreak investigations and the related programmatic evaluations/activities as public health practice since the aim is to contain the outbreak. In addition, we discussed our study with the research and ethics committee of Makerere University School of Public Health and obtained a waiver. We also sought verbal consent since our study was non intrusive, explained the risks and benefits, ensured privacy and confidentiality since personal identifiers were not used on survey tools.

## Results

This post campaign evaluation survey for yellow fever vaccination coverage was conducted between the 6^th^ and 12^th^ of May 2011. Of the 680 study participants, only 679 (99.8%) were included in our analysis. One respondent (0.2%) was excluded from further analysis on account of having an incompletely filled survey form. More than half of the respondents (54.6%) were females.

### Yellow fever vaccination coverage

Table 1 shows results of yellow fever vaccination status based on vaccination card evidence or recall disaggregated by sex and age. The overall yellow fever vaccination coverage in Pader district was 96.1% (95% CI 94.3 - 97.8). By age, the highest vaccination coverage was among respondents between 5 to 15 years, 98.0%, (95% CI 96.3 – 99.9). Vaccination coverages for children below 5 years and respondents above 15 years were 96.1% (95% CI 93.7 – 98.7) and 94.9% (95% CI 91.9 – 98.1) respectively. A higher proportion of males were vaccinated 96.9%, (95% CI 94.3 – 99.5) compared to females 95.4%, (95% CI 92.9 – 97.9). More than half of the respondents 51.6% (95% CI 47.2 – 56.1) had a yellow fever vaccination card as evidence of vaccination while 44.3% (301) reported that they had received vaccination but had no card evidence. In this survey, no invalid or fake cards were seen and a card was valid if it was signed and stamped by the relevant health authorities. Only 3.7% (25) of the respondents did not receive yellow fever vaccination.

**Table 1:** Yellow fever vaccination coverage by age and sex in Pader district

**Variables (n = 679)**	**Vaccination (Card only)**		**Vaccination ( Card+/−recall)**	
	**Frequency**	**% (95% CI)**	**Frequency**	**% (95% CI)**
**Age**				
Below 5 years	147	52.5 (46.6 – 58.3)	269	96.1 (93.7 – 98.7)
5 to 15 years	105	52.2 (45.3 – 59.2)	197	98.0 (96.3 – 99.9)
Above 15 years	101	51.0 (43.9 – 58.0)	188	94.9 (91.9 – 98.1)
**Sex**				
Female	195	50.7 (44.9 – 56.3)	355	95.4(92.9 – 97.9)
Male	158	51.3 (45.7 – 56.8)	299	96.9(94.3 – 99.5)
**Overall vaccination coverage**	353	51.6 (47.2 – 56.1)	654	96.1(94.9 – 97.8)

### Reasons for not receiving yellow fever vaccination during the campaigns

From Table 2, the main reasons for failure to receive yellow fever vaccination were: having travelled out of Pader district, distance/lack of transport to the immunization posts and sickness. Other reasons included: being at school, long waiting time and rudeness of health workers.

**Table 2:** Reasons for not receiving Yellow fever vaccination during the campaigns in Pader district

**Reasons for none vaccination**	**Frequency (%)**
Having travelled out of Pader district	10 (40.0)
Lack of transport to vaccination posts	7 (28.0)
Being sick during the vaccination campaigns	4 (16.0)
Being at school during the campaigns	2 (8.0)
Long waiting time at vaccination posts	1(4.0)
Rudeness of health workers at vaccination posts	1(4.0)

## Discussion

This post campaign survey, which was part of the outbreak response activities by the Ugandan Ministry of Health and WHO, was done to evaluate the vaccination coverage following an outbreak of yellow fever in northern Uganda. In Pader district, the overall estimate of yellow fever vaccination coverage was 96.1% with a card retention rate of 51.6%. The main reasons for not receiving vaccination during the mass campaign were: having travelled out of the district, lack of transport to vaccination posts and being sick during the campaigns. Others reasons were: being sick during the campaigns, being at school and rudeness of health workers at vaccination posts.

This overall vaccination coverage (by card and recall) in Pader district exceeded 90% and was above the desired minimum threshold coverage of 80% according to WHO [24]. This theoretically implies that a need to conduct an emergency follow up revaccination campaign was avoided since almost everybody (96.3%) was protected against yellow fever. When the desired immunization coverage is exceeded, the potential for human to human transmission reduces since a higher proportion of people at risk have circulating yellow fever antibodies. The other affected districts also reported high vaccination coverage estimates: Abim 95.4%, Agago 97.5%, Kitgum 97.8% and Lamwo 97.1%. A cluster survey done in internally displaced people’s camps in Liberia following mass yellow fever vaccination campaigns revealed comparably high coverages [25]. However, a high vaccination coverage doesn’t necessarily mean that the country is not at risk since Uganda borders South Sudan which experiences sporadic yellow fever outbreaks [17]. Secondly, yellow fever vaccination is not routinely given along with the routine EPI vaccines thus only people who travel out of the country might be protected against the disease. Indeed one case of yellow fever was reported in October 2012 in Agago district, northern Uganda and this was confirmed by the Epidemiology and Surveillance unit of Ministry of Health [26].

Our study found a difference between the survey and administrative coverage (75.9%) and this is in agreement with previous studies which suggest that the quality of administrative coverage data is unreliable [27,28]. Such data, often times over or under estimates coverage due to wrong population estimates and incomplete tallying or reporting of vaccination doses. This is compounded by poorly documented shifts in population and reliance on imprecise or outdated census data [23,25]. To our knowledge, this was the first reported cluster survey to evaluate yellow vaccination coverage in Uganda and no yellow fever outbreaks had been reported in Uganda in the last 40 years [14]. One possible reason for the discrepancy between administrative and our survey coverage is that the Ministry of Health during the immunization campaigns relied on old projected population estimates from the district population office. Secondly, the estimated target population used to calculate the administrative coverage might not have taken into account new residents, migrant workers and nomadic population.

Although, the yellow fever vaccination coverage exceeded 90% by self report (card or recall), only half of the respondents had cards as evidence for vaccination. A similar vaccination card retention rates was reported in a cluster survey for routine childhood EPI and yellow fever vaccination campaigns in West Africa [29]. In our study, we considered both card evidence and recall to estimate vaccination status. A previous survey showed that caregiver recall is highly sensitive (>90%) and fairly accurate in determining vaccination status [30]. Because of the high sensitivity of caregiver recall in estimating vaccination status, it is now acceptable practice both in developed and resource constrained countries to use caregiver recall in estimating immunization coverage [30,31]. In our survey, we validated vaccination status by recall by also asking the site of vaccination during the campaigns. The low card retention rate after a short period of mass immunization (3 months) may suggests programmatic or logistical challenges in organizing vaccination activities. It is also possible that not all people vaccinated actually received their vaccination cards. Although there were unconfirmed reports of fake yellow fever vaccination cards being sold in clinics in Kampala, the capital city of Uganda, to Ugandans travelling abroad [32], no fake vaccination cards were found during our survey.

Our study revealed that the 5–15 years age category had the highest vaccination coverage. This is probably due to the fact that this age category is easy to mobilize since most of them are in schools. Nearly fifty percent of those not vaccinated had travelled out of the district possibly to engage in other gainful activities during the vaccination exercise. Furthermore, it is possible that adults are more likely to question the rationale of vaccination than children and this may influence their decision regarding vaccination. For children below 5 years, chances of being vaccinated largely depend on adults (caregivers). Others reasons for non vaccination from our study were lack of transport, sickness, long waiting time and rudeness of health workers. This is in agreement with studies done to assess the determinants of vaccination coverage in Nairobi Kenya and Niger [33,34]. Such factors are major barriers to access of immunization and other health services by communities and thus may reinforce poor health seeking behaviors. This also reinforces the need for continuous staff motivation and regular supervision to overcome health system challenges to vaccination campaigns.

### Limitations of our survey

For children, interviews were conducted by proxy and this could have affected the accuracy of information. However, proxy interviewees were members of the same household and usually very familiar with the demographic and vaccination status of the selected person. Secondly recall bias may have been introduced since self reports were used to ascertain vaccination status for those who did not have yellow fever vaccination cards for the last campaigns. One way to minimize this is to conduct evaluation survey immediately after campaigns. This may not be feasible in Uganda which at times relies on logistical support from WHO to carry out such evaluations. Cluster lot quality assurance sampling techniques can be used to monitor coverage as soon as the campaign ends or even before its end [35]. Since we did not have the proportion of population vaccinated per strata during the campaigns, calculating overall vaccination coverage without taking this into account may have resulted in less accurate results if there were great imbalance between strata.

Our coverage estimates are only really valid for card and history since these figures are above 90%. The estimations by card only are approximately 50% so the sample size was probably too small to obtain precise estimates. We also asked about the site of vaccination or injection to validate reported vaccination status. Also, because routine EPI vaccination programs were going on in the district, bias by inadvertently attributing yellow fever immunization to another vaccine was likely. Since vaccination cards for this yellow fever immunization campaign were different from the routine EPI immunization cards, we believe such bias did not affect our data greatly. With the use of standard yellow fever survey tools, information on household size, distance from vaccination posts, religion, reasons for not having cards was not captured hence we could not draw conclusions on these variables.

## Conclusions

The mass vaccination campaigns against yellow fever in Pader district were successful since the estimated coverage exceeded the desired threshold of 80%. Consequently, mop up revaccination activities were avoided. However, active surveillance activities for early case detection of yellow fever are necessary since this area borders South Sudan which is prone to sporadic yellow fever outbreaks. Mass campaign evaluations should be done immediately or shortly after response activities in order to estimate vaccination coverage more accurately. In addition, more efforts should be put to ensure immunization card retention. In future, more vaccination posts should be established to increase access to immunization services by the population. Yellow fever survey tools could be modified to capture information on: reasons for poor card retention, religion, household size and distance from vaccination posts since such variables might affect vaccination status.

## Competing interests

Authors declare that they have no competing interests.

## Authors’ contributions

JB conceived, designed, conducted and analyzed data in this study. He also wrote the manuscript. The other authors (ER, MM, NT and IM) were jointly responsible for study concept and writing the manuscript. All authors have seen and approved the final version of the manuscript.

## Pre-publication history

The pre-publication history for this paper can be accessed here:

http://www.biomedcentral.com/1471-2458/13/202/prepub
